# BGSE-RRT*: A Goal-Guided and Multi-Sector Sampling-Expansion Path Planning Algorithm for Complex Environments

**DOI:** 10.3390/s26061837

**Published:** 2026-03-14

**Authors:** Wenhao Yue, Xiang Li, Ziyue Liu, Xiaojiang Jiang, Lanlan Pan

**Affiliations:** 1 College of Mechanical and Power Engineering, Dalian Ocean University, Dalian 116086, China; 17861237980@163.com (W.Y.); liuziyue0122@126.com (Z.L.); 15672797927@163.com (X.J.); pllan@dlou.edu.cn (L.P.); 2 College of Information Engineering, Dalian Ocean University, Dalian 116086, China

**Keywords:** path planning, RRT*, bi-tree adaptive switching, goal-guided, local multi-sector, sampling-point-guided

## Abstract

In complex ground environments, conventional RRT* often suffers from low planning efficiency and poor path quality for robot path planning. This paper proposes BGSE-RRT* (Bi-tree Cooperative, Goal-guided, low-discrepancy Sampling, multi-sector Expansion). First, BGSE-RRT* constructs a nonlinear switching probability via bi-tree cooperative adaptive switching, together with KD-Tree nearest-neighbor acceleration and multi-condition triggering, to adaptively balance global exploration and local convergence. Meanwhile, a goal-guided expansion with dynamic target binding and adaptive step size, under a multi-constraint feasibility check, accelerates the convergence of the two trees. When the goal-guided expansion becomes blocked, BGSE-RRT* generates candidate points in local multi-sector regions using a 2D Halton low-discrepancy sequence and selects the best candidate for expansion; if the multi-sector expansion still fails, a sampling-point-guided expansion is activated to continue advancing and search for a feasible path. Second, B-spline smoothing is applied to improve trajectory continuity. Finally, in five simulation environments and ROS/real-robot joint validation, compared with GB-RRT*, BI-RRT*, BI-APF-RRT*, and BAI-RRT*, BGSE-RRT* reduces planning time by up to 84.71%, shortens path length by 2.94–6.88%, and improves safety distance by 20.68–48.33%. In ROS/real-robot validation, the trajectory-tracking success rate reaches 100%.

## 1. Introduction

In recent years, autonomous driving has developed rapidly worldwide and is widely regarded as a key direction for the future of transportation systems [1]. The realization of autonomous driving relies on the coordinated operation of intelligent vehicles in perception, decision-making, path planning, and control [2]. With high-definition maps available, lane-level navigation can be achieved using high-precision localization. In this process, the objective of path planning is to generate a collision-free, kinematically constrained, continuous, and optimal path from the start to the goal [3].

In path planning, platforms such as steering vehicles [4], marine vessels [5], and robotic manipulators [6] commonly face physical constraints, including steering limits, obstacle avoidance requirements, dynamic stability, and joint limits [7]. Generating feasible paths under these constraints remains a key challenge, and a variety of solutions have been proposed [8]. However, in complex environments with obstacle-dense layouts and heavy occlusion, planners must jointly consider search efficiency, path quality, and trajectory trackability. Existing methods can be broadly categorized into four classes according to modeling and solution paradigms.

(1) Grid-based search methods (e.g., Dijkstra [9] and A* [10]) discretize the space and search over grid cells. Their accuracy depends on grid resolution: finer grids can yield smoother paths but incur higher computational complexity [11]. Some studies perform secondary optimization after search, which may be inconsistent with the original collision-avoidance result. For example, Li et al. [12] use adaptive pure pursuit to generate shorter and smoother paths based on an A* path; however, due to the discrete nature, it is still difficult to guarantee curvature continuity and trajectory trackability in a principled way, and obstacle-dense scenarios often expose a conflict between resolution and real-time performance.

(2) Optimization-based methods (e.g., artificial potential field (APF) [13]) typically rely on accurate potential-field modeling and establish a mapping between the environment model and path generation [14], showing effectiveness in handling environmental constraints such as obstacle avoidance [15]. However, under complex geometric constraints, it is often difficult to directly couple kinematic constraints with the potential-field framework, and the method is prone to local minima, leading to unstable guidance or local stagnation.

(3) Learning-based methods [16] perform planning in a data-driven manner and can be advantageous in unknown environments [17], but they depend on data coverage and prior trajectory data, and it is difficult to obtain training data that are both generalizable and asymptotically optimal. Therefore, they are often combined with conventional methods in practice. For example, Hao et al. [18] initialized a reinforcement-learning Q-table using APF on top of a sampling method; Tan et al. [19] combined a Q-learning Q-table with an A* grid structure to improve performance. Overall, learning-based outputs are often suboptimal and are mainly used to enhance local capabilities of rule-based methods; under complex environments and safety constraints, generalization stability and interpretability must be carefully balanced.

(4) Sampling-based planners (e.g., RRT and PRM) [20] provide probabilistic completeness and good scalability [21] and are widely used for constrained path planning tasks [22]. The resulting path shape is largely determined by the connection/rewiring strategy [23]; thus, many studies improve connection schemes to satisfy specific constraints, such as cubic spline interpolation [24], circular-arc methods [25], quadratic programming [26], Bézier-curve methods [27], and bio-inspired methods [28]. Dubins curves [29] enforce curvature via a minimum turning radius but typically require specified boundary poses and headings. Some studies introduce kinematic constraints during expansion or connection [30], which may reduce the success rate of initial connectivity; moreover, inconsistency between the terminal heading of one segment and the initial heading of the next can lead to poor trajectory trackability, which is insufficiently addressed in part of the literature.

Moreover, the computational efficiency of sampling-based planners in complex environments strongly depends on the sampling distribution and the quality of heuristic guidance. Related work reduces ineffective expansions mainly from search strategies and sampling strategies [31]. For search strategies, bidirectional planning and bi-tree search structures are used to accelerate feasible-solution discovery. For example, FHQ-RRT [32] speeds up high-quality path generation through strategy design and GD-RRT* [33] improves initial-solution quality and optimization efficiency by combining Gaussian sampling and deep policies; however, multi-tree parallel methods may still produce infeasible paths at the merging stage [34]. For sampling strategies, shrinking the sampling space and pruning are common. Informed-RRT* [35] restricts sampling to an ellipsoidal subset, RRT-FN [36] introduces pruning to control tree size, and DBVSB-P-RRT* [37] improves adaptability in complex environments via adaptive biased sampling, variable step size, and pruning. Nevertheless, many of these improvements focus on a single aspect (e.g., sampling space, tree size, or bias form). In obstacle-dense and narrow-passage scenarios, planners may still suffer from failed goal guidance, blocked expansion, and frequent collision rejection, causing fluctuations in convergence efficiency and path quality; meanwhile, curvature continuity and trajectory trackability often require coordinated search mechanisms.

Beyond these mainstream methods, advanced path planning methods developed in recent years for specific tasks and complex physical constraints also merit attention. For example, Bahwini et al. [38] addressed needle insertion path planning under soft-tissue deformation by establishing a temperature-field model based on bioheat transfer, thereby accounting for the effect of soft-tissue deformation on path generation. Zhong et al. [39] proposed a cellular-neural-network-based method for optimal robot path planning, which enabled real-time path generation through neural dynamics without requiring global search. Building on this work, Hills and Zhong [40] further introduced a thermal modeling method that incorporated the heat conduction process into real-time robot path planning, thereby enhancing responsiveness to changes in dynamic environments. In the field of aerial robots, Zhou et al. [41] proposed a crossover-recombination-based globally optimal brain storm optimization algorithm that combined cubic B-splines, constraint handling, and swarm intelligence optimization for multi-constraint UAV path planning. These studies indicate that path planning has expanded from traditional geometric obstacle avoidance to more complex scenarios involving soft-tissue deformation, thermal-field modeling, neural dynamics, and multi-constraint flight optimization. However, such methods generally rely on specific physical models, tasks, or optimization frameworks, and therefore remain clearly distinct from the tree-based incremental sampling planning problem in known static complex ground environments considered in this study.

In summary, improving RRT* in complex environments faces the following key challenges: unstable and non-adaptive goal guidance that induces ineffective expansions and search stagnation; fixed goal bias that easily triggers local oscillation near obstacles; occluded structures that require more directional and uniform candidate generation to reduce repeated trial and error; and polyline connection/rewiring alone that struggles to achieve both optimality and curvature continuity, thereby degrading trajectory trackability.

To address these issues, this paper proposes BGSE-RRT*, a bi-tree cooperative and multi-mechanism integrated path planning algorithm. Our target problem is 2D planar path planning and trackable trajectory generation for ground mobile robots under known-map constraints, where “complex environments” mainly refer to geometric complexity such as obstacle-dense layouts, heavy occlusion, and narrow passages. The main contributions are as follows: (1) a nonlinear switching probability combined with KD-Tree acceleration and multi-condition triggering enables adaptive balancing between global exploration and local convergence, improving expansion efficiency; (2) a goal-guided expansion accelerates bi-tree convergence, and when expansion becomes blocked, 2D Halton low-discrepancy sampling within multi-sector regions generates candidates and selects the best to enhance obstacle avoidance; and (3) if the multi-sector expansion still fails, a sampling-point-guided expansion continues advancing to find a feasible path, and finally B-spline post-processing improves path continuity.

The remainder of this paper is organized as follows: Section 2 introduces BI-RRT*, APF-RRT*, and BI-APF-RRT* baselines and clarifies the comparison scope with sampling-based planners. Section 3 presents BGSE-RRT*. Section 4 describes the experimental environments and evaluates BGSE-RRT* across multiple scenarios, with comparisons to GB-RRT*, BI-RRT*, BI-APF-RRT*, and BAI-RRT*. Section 5 concludes the paper.

## 2. Related Work

To address the difficulty of jointly achieving path quality, expansion efficiency, and adaptability in complex environments and to ensure representative and reproducible comparisons, this section selects BI-RRT* [30], APF-RRT* [8], and BI-APF-RRT* [24] as baselines. Specifically, BI-RRT* improves feasible-path discovery via bidirectional expansion; APF-RRT* uses potential-field information to provide heuristic guidance for tree expansion; and BI-APF-RRT* further integrates a “bi-tree structure + potential-field guidance,” which is the closest to our approach. However, BI-APF-RRT* may still encounter blocked expansion or local oscillation in complex environments, leading to slower convergence and fluctuating path quality; therefore, using it as a strongly related baseline better validates the improvements in BGSE-RRT* in complex environments.

### 2.1. BI-RRT*

**Figure 1:**
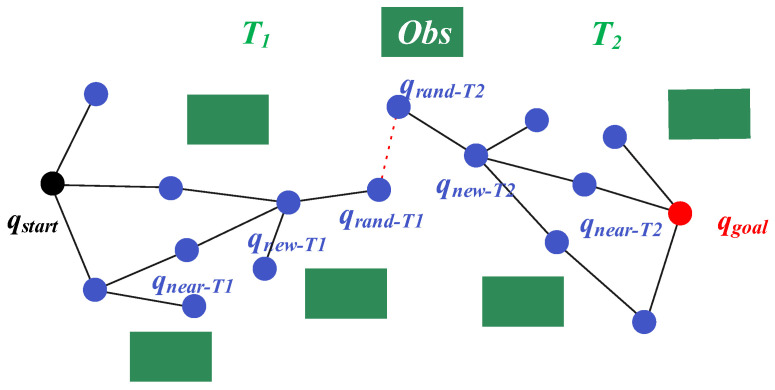
Schematic diagram of the BI-RRT* algorithm.

RRT* incrementally expands a single search tree outward, whereas BI-RRT* constructs two trees from the start and the goal simultaneously. By alternating expansions and repeatedly attempting to connect the two trees, the search progresses them toward each other from both ends and they merge earlier. Benefiting from this bidirectional growth mechanism, BI-RRT* typically achieves higher expansion efficiency than single-tree methods and can obtain a feasible path faster. However, in complex environments, BI-RRT* may still exhibit slow convergence and unstable path quality. The principle of BI-RRT* is illustrated in Figure 1.

### 2.2. APF-RRT*

**Figure 2:**
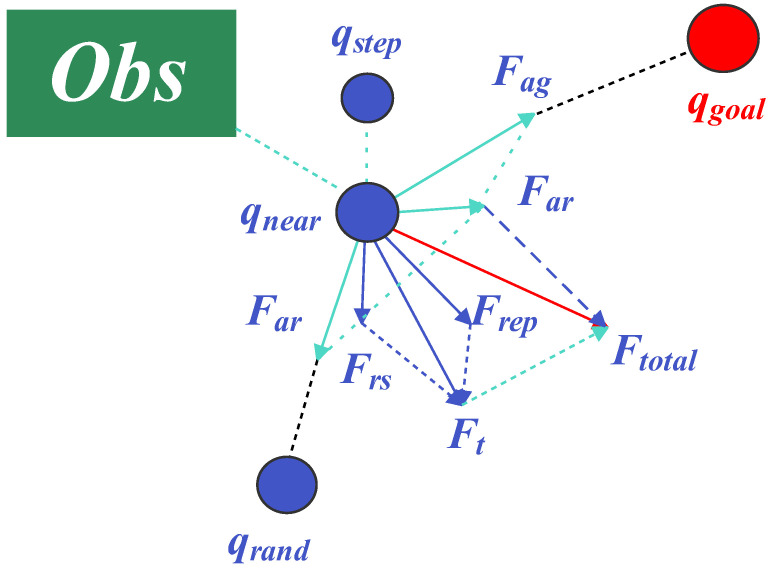
Schematic diagram of the APF-RRT* algorithm.

APF-RRT* integrates artificial potential field (APF) guidance into the expansion process of RRT*. It applies attraction to the goal $q_{goal}$ and the random sample $q_{rand}$, constructs repulsion from obstacles, and uses the resultant force $F_{total}$ to guide new-node growth so that expansions move toward the goal while avoiding collisions. The procedure is as follows: initialize the tree *T* at $q_{start}$; sample $q_{rand}$; find the nearest node $q_{near}$; compute $F_{total}$ by combining attraction and repulsion; and generate a new node $q_{new}$ along this direction with step size *s*. If the segment is collision-free, add $q_{new}$ and perform connection and rewiring by selecting the parent with the minimum accumulated cost in the neighborhood; otherwise, discard the node and resample until termination. APF-RRT* preserves the rewiring capability of RRT* while improving the goal guidance and near-obstacle avoidance tendency; however, it is prone to local minima in complex environments, and potential-field evaluation (e.g., near-obstacle distance) introduces extra computational burden, affecting stability. The principle of APF-RRT* is illustrated in Figure 2.

### 2.3. BI-APF-RRT*

**Figure 3:**
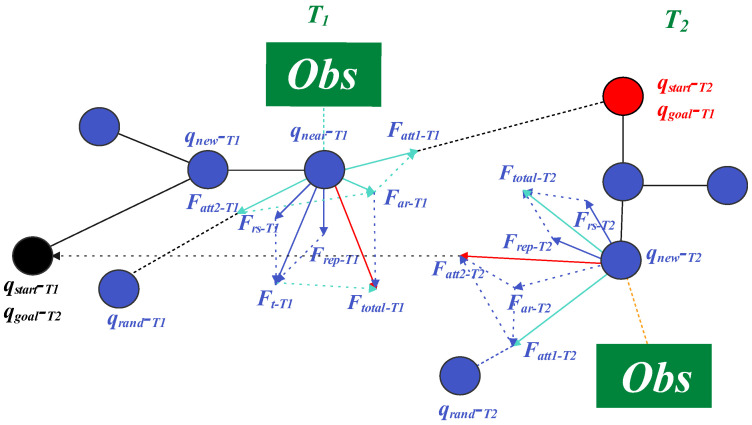
Schematic diagram of the BI-APF-RRT* algorithm.

BI-APF-RRT* grows two search trees $T_{s}$ and $T_{g}$ rooted at $q_{start}$ and $q_{goal}$, respectively, and alternates expansion between them. The algorithm samples $q_{rand}$, selects the nearest node $q_{near}$ in the current tree, and forms the resultant guidance force $F_{total}$ as a weighted sum of the goal attraction term, the sampling-guidance term, and the obstacle repulsion term. Starting from $q_{near}$, it generates a new node $q_{new}$ along the normalized direction of $F_{total}$ with a fixed step size *s*. If $q_{new}$ is collision-free, it is inserted into the current tree and RRT* rewiring is performed to reduce path cost. When the distance between the nearest nodes of the two trees is below a threshold, a collision-free connection is attempted; if successful, the path is backtracked and returned. Built on the asymptotically optimal rewiring mechanism of RRT, BI-APF-RRT* combines bidirectional expansion and potential-field guidance to enhance goal guidance and obstacle avoidance, thereby obtaining feasible paths faster; nevertheless, in complex environments it may still suffer from blocked expansion and local oscillation, resulting in slower convergence and unstable path quality. The principle of BI-APF-RRT* is illustrated in Figure 3.

### 2.4. Relationship to Representative SOTA Sampling-Based Planners and Comparison Scope

It should be noted that sampling-based motion planning does not have a single “universal SOTA,” since performance strongly depends on task dimensionality, environment structure, collision-checking cost, and whether dynamic obstacles exist. Surveys and benchmark studies often regard BIT*, FMT*, RRT#, and RRTX (for dynamic replanning) as representative planners with clear theoretical guarantees and broad adoption. BIT* [42] uses batch sampling with implicit random geometric graph search, offering advantages in asymptotic optimality and heuristic contraction; FMT* [43] reduces unnecessary collision checks via pre-sampling and lazy connections; RRT# [44] improves interpretability of optimality convergence through vertex-consistency maintenance; and RRTX [45] provides incremental replanning capability for changing environments. In contrast, BGSE-RRT* is a structural enhancement within the incremental tree-search framework of RRT*: it improves initial connectivity efficiency and path quality in complex static environments through bi-tree cooperative adaptive switching, goal-guided expansion, and local multi-sector low-discrepancy sampling with best-candidate expansion and ensures trajectory continuity via B-spline post-processing.

Following the fairness principle of “same paradigm, same constraints, and same collision-checking model,” our experiments primarily select representative RRT*-family variants that directly correspond to our improvement line as baselines in order to isolate and verify gains brought by the proposed structural mechanisms. Meanwhile, we provide a comparative discussion of BIT*/FMT*/RRT#/RRTX in terms of paradigm differences, applicable conditions, and expected advantages/limitations, thereby clarifying the boundary of our contribution and the scope of comparison.

## 3. Principle of the BGSE-RRT* Algorithm

Although BI-APF-RRT* optimizes the planning time and path quality of RRT* to a certain extent, it still struggles to effectively balance path quality, expansion efficiency and environmental adaptability. To address this issue, the BGSE-RRT* algorithm proposed in this paper realizes adaptive coordination of bi-tree expansion through bi-tree adaptive switching; accelerates the convergence of the two trees via goal-guided expansion and switches to local multi-sector expansion for comprehensive optimal selection and advancement if the expansion is blocked; activates sampling-point-guided expansion to continue advancing and find a feasible path when the multi-sector expansion fails; and finally adopts B-spline to improve trajectory continuity.

### 3.1. Bi-Tree Cooperative Adaptive Switching Strategy

Aimed at the problem that the traditional BI-RRT* usually adopts fixed goal bias, which is prone to invalid guidance and expansion stagnation in complex environments, this paper takes the nearest connectable distance between the two trees as the feedback quantity and combines the nonlinear switching probability, KD-Tree nearest-neighbor search and multi-condition triggering mechanism to realize the adaptive balance between global exploration and local convergence.

(1) Nonlinear Switching Probability Model

The switching probability is defined as a piecewise function:

(1) $$\Omega_{s}\left(D\right)=\left\{\begin{array}{c} \Omega_{\max}-\left(\Omega_{\max}-\Omega_{\min}\right){\left(\frac{D}{D_{th}}\right)}^{2},\;0\leq D\leq D_{th}\\ \Omega_{\min},\;D>D_{th} \end{array}\right.$$

where *D* is the minimum distance from the current expansion node $q_{curr}$ to the connectable nodes in the opposite tree, $D_{th}$ is the switching threshold ($D_{th}>0$), and $\Omega_{\max}$ and $\Omega_{\min}$ are the upper and lower limits of the switching probability ($0<\Omega_{\min}<\Omega_{\max}<1$). When $D\ll D_{th}$, $\Omega_{s}\left(D\right)$ increases to $\Omega_{\max}$ to enhance the convergence of the two trees; when $D>D_{th}$, $\Omega_{s}\left(D\right)$ decreases to $\Omega_{\min}$ to suppress excessive goal bias and maintain exploration capability.

(2) KD-Tree Nearest-Neighbor Search Acceleration

To avoid a nearest-neighbor search in each expansion, a KD-Tree index is established for the node coordinates of the opposite tree. When calculating the distance *D* or performing switching judgment, the current expansion node $q_{curr}$ is taken as the query point to execute nearest-neighbor search on the KD-Tree of the opposite tree, which reduces the complexity of nearest-neighbor search from linear scanning to $O(\log M_{term})$, where $M_{term}$ is the number of nodes in the opposite tree.

(3) Distance Pruning and Collision Detection Optimization

The current optimal connectable distance $d_{min}$ is maintained for the candidate nearest-neighbor set. For the candidate node $q_{i}$, the Euclidean distance $d(q_{curr},q_{i})$ is calculated first:

If ($d\left(q_{curr},q_{i}\right)\geq d_{min}$), the candidate cannot update the current optimal value, and the collision detection is skipped;

If ($d\left(q_{curr},q_{i}\right)<d_{min}$), the collision detection of the connecting line segment is executed only at this time, and $d_{min}=d\left(q_{curr},q_{i}\right)$ is updated if there is no collision.

Finally, $D=d_{min}$ is taken, and the corresponding node is regarded as the “nearest connectable node”.

(4) Multi-condition Triggering Logic

To avoid premature switching or long-term non-triggering, the following triggering criterion is constructed:

(2) $$Tr=\left\{\begin{array}{c} True,\;\left\{\begin{array}{c} t_{iter}\geq t_{iter,min}\\ D<D_{th}\\ \xi <\Omega_{s}\left(D\right) \end{array}\right.\\ False,\;otherwise \end{array}\right.$$

where $t_{iter}$ is the current number of iterations, $t_{iter,min}$ is the minimum effective exploration iteration threshold, and ξ∼U(0,1) is a uniformly random number. The three conditions are used to suppress invalid switching in the initial stage, limit the action range of switching, and adaptively adjust the strength of goal guidance according to $\Omega_{s}\left(D\right)$, thereby improving the stability of the switching mechanism.

(5) Rate of Change Analysis

The derivative of Equation (1) in the interval ($0\leq D\leq D_{th}$) is obtained as:

(3) $$\frac{d\Omega_{s}\left(D\right)}{dD}=-2\left(\Omega_{\max}-\Omega_{\min}\right)\frac{D}{D_{th}^{2}},\;0\leq D\leq D_{th}$$

When *D* decreases, $\Omega_{s}\left(D\right)$ increases to $\Omega_{\max}$, thus enhancing goal guidance in the short-distance stage and accelerating the state switching when the two trees approach each other. When $D>D_{th}$, $\Omega_{s}\left(D\right)=\Omega_{\min}$ and $d\Omega_{s}/dD=0$, and the two trees cover the unknown space in a relatively independent expansion mode; when $D_{th}/2<D\leq D_{th}$, $\Omega_{s}\left(D\right)$ increases gradually to conduct a tentative approach with a low probability; when $D\leq D_{th}/2$, $\Omega_{s}\left(D\right)$ increases and approaches $\Omega_{\max}$, realizing the rapid convergence of the two trees.

**Figure 4:**
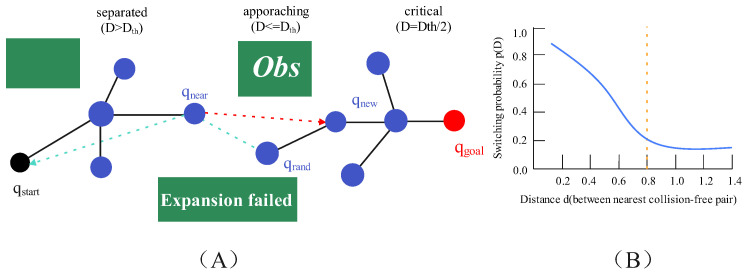
Schematic diagram of the bi-tree cooperative adaptive switching strategy.

In summary, this strategy enables the strength of goal bias to be adaptively adjusted with the connectable distance between trees and the search stage through nonlinear switching probability, KD-Tree nearest-neighbor acceleration and multi-condition triggering; provides trigger conditions and switching logic for subsequent expansion strategies; and thus reduces invalid expansion. The principle of bi-tree cooperative adaptive switching is shown in Figure 4.

### 3.2. Goal-Guided Expansion Strategy

Aimed at the problem that traditional goal-biased sampling is prone to generating a large number of invalid expansions in complex scenarios, which further leads to search stagnation, this strategy introduces dynamic target binding, adaptive step size and a multi-constraint feasibility check to improve the probability of effective expansions toward the target, thus accelerating the convergence of the two trees.

(1) Dynamic Target Binding

Let *L* be the distance between the nearest nodes of the two trees and $L_{th}$ be the coordination threshold. When $L>L_{th}$ (separate exploration), the start tree $T_{init}$ takes the goal $q_{goal}$ as the expansion target and the goal tree $T_{term}$ takes the start $q_{init}$ as the expansion target to maintain global exploration of the unknown space; when $L\leq L_{th}$ (cooperative convergence), the target of $T_{init}$ is switched to the nearest connectable node $q_{near}$ in $T_{term}$, and $T_{term}$ is bound to the corresponding nearest connectable node in $T_{init}$ at the same time, prompting the two trees to converge rapidly along the shortest connectable direction.

(2) Goal-guided Expansion and Adaptive Step Size

Given the current expansion point $q_{curr}$ and the expansion target $q_{tar}$, the original direction vector is defined as:

(4) $$v_{raw}=q_{tar}-q_{curr}$$

and the unit expansion direction is obtained by normalization:

(5) $$dir=\frac{v_{raw}}{∥v_{raw}∥}\;(∥v_{raw}∥\;\neq 0)$$

where $∥v_{raw}∥$ is the Euclidean norm. To enable the step size to adaptively change with the local obstacle congestion degree, an environmental complexity index ζ∈[0,1] is introduced in this paper to describe the “local congestion level” of the neighborhood of the current expansion point. Specifically, a local evaluation neighborhood $N(q_{curr},R_{c})$ is taken with $q_{curr}$ as the center ($R_{c}$ is a fixed evaluation scale with the same dimension as the planning scale), and two types of information including obstacle proportion and near-obstacle safety distance are counted in this neighborhood: let *K* detection points $x_{k}$ be distributed in $N(q_{curr},R_{c})$, then the obstacle proportion is defined as $\rho_{occ}=\left(1/K\right)\sum I\left[x_{k}\in Obs\right]$; at the same time, the normalized term of the nearest obstacle safety distance is calculated as $\rho_{clr}=1-\mathrm{clip}\left(D_{min}\left(q_{curr},Obs\right)/R_{c},0,1\right)$. Finally, the environmental complexity index is obtained as:

(6) $$\zeta =\mathrm{clip}\left(w_{occ}\cdot \rho_{occ}+w_{clr}\cdot \rho_{clr},0,1\right),w_{occ}+w_{clr}=1$$

A larger ζ indicates a more crowded local area or a closer distance to obstacles, while a smaller ζ indicates a more open local area. Based on ζ, the expansion step size is mapped to a continuous function δ(ζ) within the interval $\left[\delta_{min},\delta_{max}\right]$: a larger step size is adopted in simple environments (ζ<a) to accelerate expansion; a smaller step size is adopted in complex environments (ζ≥b) to enhance obstacle avoidance; and linear interpolation is used for compromise processing in the transition region (a≤ζ<b). The candidate new node is:

(7) $$q_{new}=q_{curr}+\delta \left(\zeta \right)dir$$

If the current expansion tree is switched to the other tree, the current node of this tree is taken as $q_{curr}$, and the recalculation is performed according to Equations (4)–(7).

(3) Expansion Deviation Angle and Basis for Direction Correction

To quantify the consistency between the local expansion direction and the global target direction, the expansion deviation angle is introduced:

(8) $$\Delta \phi =\arccos\left(\frac{\left(q_{tar}-q_{curr}\right)\cdot \left(q_{goal}-q_{curr}\right)}{∥q_{tar}-q_{curr}∥∥q_{goal}-q_{curr}∥}\right)$$

where $q_{goal}$ denotes the global target of the current expansion tree. A smaller Δϕ indicates that the expansion direction is closer to the global connectable direction and the path redundancy is lower; a larger Δϕ indicates an obvious deviation, which triggers local multi-sector expansion for direction correction. If the denominator is 0, Δϕ=0 is set or the calculation is skipped.

(4) Expansion Success Score

An expansion success score based on step size and safety distance is constructed:

(9) $$P_{succ}=\alpha \cdot e^{-\beta \cdot \delta }+\gamma \frac{D_{obs}\left(q_{add},O\right)}{D_{max}\left(O\right)},\alpha \in \left[0,1\right],\alpha +\gamma =1$$

where α, β, and γ are adjustment coefficients; $D_{obs}\left(q_{add},O\right)$ is the distance from the new node to obstacles; and $D_{max}\left(O\right)$ is the maximum value of this statistic in the scenario. Equation (9) shows that an excessively large step size will reduce the success score, while a larger average distance to obstacles will increase the success score. Therefore, introducing the environmental complexity ζ into δ can adaptively balance the expansion speed and obstacle avoidance safety.

It should be emphasized that ζ is an environmental quantity obtained online by local geometric/collision detection statistics, rather than a manual parameter tuning for a single scenario; it maintains a large step size in open areas to improve exploration efficiency and automatically shortens the step size in crowded/near-obstacle areas to reduce invalid expansions, thus improving the stability and transferability of goal-guided expansion. Moreover, $\mathrm{EvaluateComplexity}(q_{curr},Obs)$ in Algorithm 1 is used to calculate the environmental complexity index ζ of the current node neighborhood; AdaptiveStep(ζ) maps ζ to the step size δ(ζ), thereby maintaining a large step size in open areas and automatically shortening the step size in crowded/near-obstacle areas to improve the probability of feasible expansion.

In summary, this strategy promotes the cooperative convergence of the two trees through dynamic target binding and reduces invalid expansions by combining adaptive step size and a multi-constraint feasibility check, thus rapidly improving the connection efficiency under the constraint of safety distance; if the expansion fails, it switches to local multi-sector to generate new feasible expansion directions. The flow of goal-guided expansion is shown in Algorithm 1.
**Algorithm 1** Goal-guided expansion.  1:$L\leftarrow \mathrm{DualTree}\;\mathrm{MinDistance}(T_{cur},T_{opp})$  2:if $L>L_{th}$ then  3:      *if Root* $\left(T_{cur}\right)\;==\;q_{init}\;\mathrm{then}\;q_{tar}\leftarrow q_{goal}\;\mathrm{else}\;q_{tar}\leftarrow q_{init}$  4:else  5:$q_{tar}\leftarrow \mathrm{KDTreeNearest}\left(T_{opp},q_{curr}\right)$  6:$v_{raw}\leftarrow q_{tar}-q_{curr}$  7:if $∥v_{raw}∥\;=\;0$ then return null  8:$\mathrm{dir}\leftarrow v_{raw}/∥v_{raw}∥$  9:$\zeta \leftarrow \mathrm{Evaluate}\;\mathrm{Complexity}(q_{curr},\mathrm{Obs})$10:δ←AdaptiveStep(ζ)11:$q_{new}\leftarrow q_{curr}+\delta \cdot \mathrm{dir}$12:If $\mathrm{Feasible}\;\mathrm{Segment}(q_{curr},q_{new},\mathrm{Obs},{\mathrm{Dist}}_{safe},\mathrm{bounds})$ then13:      $\mathrm{AddNode}(T_{cur},q_{new},\mathrm{parent}=q_{curr})$14:      $\mathrm{KDTree}\;\mathrm{Insert}(T_{cur},q_{new})$15:      $\mathrm{Update}\;\mathrm{DualTree}\;\mathrm{Distance}(T_{cur},T_{opp})$16:      return $q_{new}$17:return null

### 3.3. Local Multi-Sector Refined Expansion

When the goal-guided expansion fails, this strategy takes the direction from the current node $Q_{0}=\left(X_{0},Y_{0}\right)$ to the target point $Q_{goal}=\left(X_{g},Y_{g}\right)$ as the center, constructs multi-sector partitions in its forward half-plane, generates candidate points combined with a 2D Halton low-discrepancy sequence, and selects the optimal expansion node through multi-objective comprehensive evaluation, thus improving the obstacle avoidance capability.

The azimuth angle is:

(10) $$\varphi_{0}=atan2\left(Y_{g}-Y_{0},X_{g}-X_{0}\right)$$

In the polar coordinate system of $Q_{0}$, a core sector centered on the target direction is constructed:

(11) $$\Psi_{core}=\left[\varphi_{0}-\theta_{0},\varphi_{0}+\theta_{0}\right]$$

where $\theta_{0}$ is the half-angle of the core sector. The denser the obstacles are, the smaller the value of $\theta_{0}$ is, so as to improve the sampling concentration in the target direction and reduce invalid attempts in the blocked direction. *M* groups of symmetric sectors are constructed on both sides of the core sector, and the angle interval of the *m*-th group is:

(12) $$\Psi_{m}=\left[\varphi_{0}+\theta_{m-1},\varphi_{0}+\theta_{m}\right]\cup \left[\varphi_{0}-\theta_{m},\varphi_{0}-\theta_{m-1}\right],m=1,2,\ldots ,M$$

with:

(13) $$\theta_{m}=\theta_{0}+m\Delta \theta$$

where Δθ is the angle increment. In each sector sub-region, a 2D Halton sequence $v_{j}=\left(v_{j1},v_{j2}\right)\in \left(0,1\right)$ is used to generate candidate points $Q_{j}=\left(X_{j},Y_{j}\right)$. Let $u=v_{j1}$, then the polar radius and polar angle of the candidate points are:

(14) $$\rho_{j}=\sqrt{\rho_{min}^{2}+u\left(R_{max}^{2}-\rho_{min}^{2}\right)}$$

(15) $$\varphi_{j}=\varphi_{min,m}+v_{j2}\left(\varphi_{max,m}-\varphi_{min,m}\right)$$

Thus:

(16) $$X_{j}=X_{0}+\rho_{j}\cos\varphi_{j},\;Y_{j}=Y_{0}+\rho_{j}\sin\varphi_{j}$$

where $R_{max}$ is the upper limit of the expansion step size, $\rho_{min}$ is the inner safety radius, and $\left[\varphi_{min,m},\varphi_{max,m}\right]$ is the sector angle boundary.

Feasibility constraints are imposed on the candidate points:

(17) $$\mathrm{Dist}\left(Obs,Q_{j}\right)\geq {\mathrm{Dist}}_{safe},\mathrm{Dist}\left(Q_{goal},Q_{j}\right)\leq {\mathrm{Dist}}_{goal,max}+\beta R_{max},\beta \in \left(0,1\right)$$

where $\mathrm{Dist}(Obs,Q_{j})$ denotes the distance from the candidate point to the nearest obstacle, ${\mathrm{Dist}}_{safe}$ is the safety distance threshold, and $\mathrm{Dist}(Q_{goal},Q_{j})$ is the distance from the candidate point to the target point. The candidate point can be used as an expansion node only after passing the subsequent line segment collision detection, where

(18) $${\mathrm{Dist}}_{goal,max}=\mathrm{Dist}\left(Q_{0},Q_{goal}\right)+R_{max}$$

To adaptively adjust the exploration intensity of different sectors, an obstacle density index is introduced:

(19) $$\eta_{m}=\frac{1}{A_{m}}\sum_{Obs\in O_{m}}\exp\left(-\frac{\mathrm{Dist}(Q_{0},Obs)}{R_{0}}\right)$$

where $A_{m}$ is the area of the sector, $O_{m}$ is the set of obstacles in the sector, and $R_{0}$ is the scale coefficient; a larger $\eta_{m}$ indicates a more crowded area, and thus the sampling allocation and expansion priority are reduced.

The allocated number of sampling points is constructed as:

(20) $$N_{m}\propto 1/\left(1+\eta_{m}\right)$$

To screen expansion nodes that take into account safety, linearity, goal guidance and smoothness from the candidate points, a multi-objective comprehensive evaluation function is constructed:

(21) $$F\left(Q_{j}\right)=\omega_{1}F_{s}\left(Q_{j}\right)+\omega_{2}F_{l}\left(Q_{j}\right)+\omega_{3}F_{g}\left(Q_{j}\right)+\omega_{4}F_{sm}\left(Q_{j}\right)$$

where $\omega ={\left[\omega_{1},\omega_{2},\omega_{3},\omega_{4}\right]}^{T}$ is the weight vector, and each sub-item is defined as follows:

(1) Safety Score

(22) $$F_{s}\left(Q_{j}\right)=\frac{\mathrm{Dist}(Obs,Q_{j})}{{\mathrm{Dist}}_{max}}$$

where

(23) $${\mathrm{Dist}}_{max}=\max_{Q_{j}\in Q_{m}}\mathrm{Dist}\left(Obs,Q_{j}\right)$$

if ${\mathrm{Dist}}_{max}=0$, ${\mathrm{Dist}}_{max}\leftarrow {\mathrm{Dist}}_{max}+\varepsilon$ is set to avoid division by zero. ${\mathrm{Dist}}_{max}$ takes the maximum value over the entire set of candidate points.

(2) Linearity Score

(24) $$F_{l}\left(Q_{j}\right)=\frac{1}{1+\mathrm{Dist}\left(Q_{j},L_{std}\right)/D_{ref}}$$

where $L_{std}$ is the reference line from $Q_{0}$ to $Q_{goal}$ and $D_{ref}=R_{max}$ is the normalized distance.

(3) Goal Guidance Score

(25) $$F_{g}\left(Q_{j}\right)=\frac{1}{1+\mathrm{Dist}\left(Q_{j},Q_{goal}\right)/D_{ref}}$$

(4) Smoothness Score

(26) $$F_{sm}\left(Q_{j}\right)=\frac{1}{1+\varphi_{rot}\left(Q_{j}\right)}$$

where the turning angle is given by the included angle among the parent node $Q_{par}$, the current node $Q_{0}$ and the candidate point $Q_{j}$:

(27) $$\varphi_{rot}\left(Q_{j}\right)=\arccos\left(\frac{{\vec{V}}_{1}\cdot {\vec{V}}_{2}}{∥{\vec{V}}_{1}∥\;∥{\vec{V}}_{2}∥}\right)$$

${\vec{V}}_{1}=Q_{0}-Q_{par}$ is the direction vector from the parent node to the current node, ${\vec{V}}_{2}=Q_{j}-Q_{0}$ is the direction vector from the current node to the candidate point, and $∥\vec{V}∥$ is the Euclidean norm of the vector. If the denominator is 0, $\varphi_{rot}=0$ is set, and interval truncation is performed on the cosine term to ensure numerical stability. Finally, the candidate point that maximizes $F(Q_{j})$ is selected as the expansion node.

In terms of weight determination and numerical setting, the priority of “safety > goal guidance > (linearity ≈ smoothness)” is mapped to:

(28) $$p=\left[p_{s},p_{l},p_{g},p_{sm}\right]=\left[4,1.5,3,1.5\right]$$

and the normalized weight is obtained as:

(29) $$\omega_{k}=\frac{p_{k}}{\sum_{i=1}^{4}p_{i}},\omega_{k}\geq 0,\sum_{k=1}^{4}\omega_{k}=1$$

A unified weight vector is adopted in the experiments of this paper:

(30) $$\omega ={\left[0.4,0.15,0.3,0.15\right]}^{T}$$

corresponding to safety, linearity, goal guidance and smoothness, respectively. For different scenario preferences, *p* can be adjusted on the premise of keeping the normalization rule unchanged.

**Figure 5:**
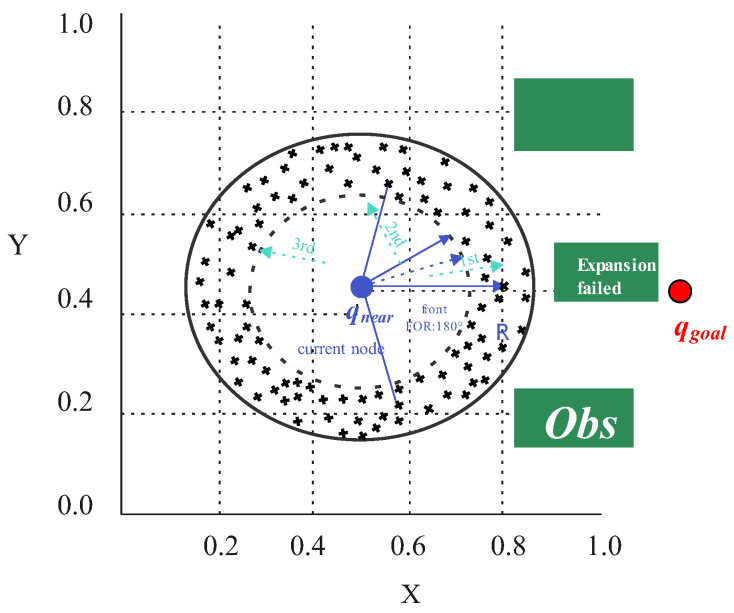
Schematic diagram of local multi-sector refined expansion.

This strategy enhances the effective exploration of the forward space through “multi-sector partitioning –low-discrepancy sampling –multi-objective screening”, and complements the goal-guided expansion, thus suppressing redundancy and alleviating the stagnation of unidirectional expansion. When the local multi-sector expansion still fails, the sampling-point-guided expansion is triggered to provide continuous and feasible detour directions. The principle of local multi-sector refined expansion is shown in Figure 5.

### 3.4. Sampling-Point-Guided Expansion Strategy

When the local multi-sector expansion still fails, this paper adopts an advancement mode of “random sampling point guidance + direction normalization + fixed step size” and performs a feasibility check under the constraints of collision, safety distance and boundary to continue advancing and to improve the probability of finding a feasible path.

Let the current expansion node be $Q_{0}=\left(x_{0},y_{0}\right)$ and the random sampling point be $Q_{r}=\left(x_{r},y_{r}\right)$, then the original expansion direction vector is:

(31) $$\lambda_{\mathrm{raw}}=Q_{r}-Q_{0}$$

To eliminate the influence of sampling distance on expansion stability, the normalized direction vector is defined as:

(32) $$\lambda =\left\{\begin{array}{c} 0,\;∥\lambda ∥=0\\ \frac{\lambda }{∥\lambda ∥},\;\mathrm{else} \end{array}\right.$$

When ∥λ∥=0, the current expansion is terminated to avoid generating duplicate nodes; otherwise, a new node is generated with a fixed step size *s*:

(33) $$Q_{\mathrm{new}}=Q_{0}+\lambda s$$

Let $Q_{\mathrm{new}}=\left(x_{\mathrm{new}},y_{\mathrm{new}}\right)$ and define the expansion line segment $L=\bar{Q_{0}Q_{\mathrm{new}}}$. To ensure feasibility and safety, collision detection is performed on the line segment L, and the minimum safety distance and boundary constraints are judged:

(34) $$\Gamma =\left\{\begin{array}{c} True\;\;\mathrm{if}\left\{\begin{array}{c} F_{\mathrm{free}}\left(L\right)=True\\ \min_{Q\in L}Dist\left(Obs,Q\right)>Dist_{\mathrm{safe}}\\ X_{\min}\leq x_{\mathrm{new}}\leq X_{\max},\;Y_{\min}\leq y_{\mathrm{new}}\leq Y_{\max} \end{array}\right.\\ False\;\;\mathrm{else} \end{array}\right.$$

where $F_{\mathrm{free}}\left(L\right)$ indicates that the line segment L has no intersection with the obstacle set Obs and $\left[X_{\min},X_{\max}\right]$, $\left[Y_{\min},Y_{\max}\right]$ are the planning space boundaries. When Γ=True, $Q_{\mathrm{new}}$ is added to the search tree; otherwise, the expansion is discarded.

To facilitate subsequent path quality evaluation and local rewiring, the safety and target connectivity are recorded synchronously when the node is inserted:

(35) $$Tre\leftarrow Tre\cup \left\{\begin{array}{cc} n_{i} & =Idx(Q_{\mathrm{new}})\\ n_{\mathrm{par}} & =Idx(Q_{0})\\ d_{\min} & =\min_{Q\in L}D_{\mathrm{obs}}\left(Q\right)\\ d_{\mathrm{goal}} & =∥Q_{\mathrm{new}}-Q_{\mathrm{goal}}∥ \end{array}\right\}$$

where $n_{i}$ is the index of the new node and $n_{\mathrm{par}}$ is the index of the parent node.

In summary, as a fallback strategy when both goal-guided expansion and local multi-sector expansion are blocked, this strategy realizes stable advancement through direction normalization and fixed step size and screens safe nodes by combining collision detection, safety distance and boundary constraints, providing information support for subsequent reconnection and path evaluation; its flow is shown in Algorithm 2.
**Algorithm 2** Sampling-point-guided expansion.  1:$\lambda_{raw}\leftarrow Q_{r}-Q_{0}$  2:if $∥\lambda_{raw}∥<\epsilon$ then return null  3:$Q_{new}\leftarrow Q_{0}+s\cdot (\lambda_{raw}/∥\lambda_{raw}∥)$  4:if $Q_{new}.x\notin \left[X_{min},X_{max}\right]$ or  5:$Q_{new}.y\notin \left[Y_{min},Y_{max}\right]$ then return null  6:$L\leftarrow \mathrm{Segment}(Q_{0},Q_{new})$  7:if not CollisionFree(L) then return null  8:$d_{min}\leftarrow \mathrm{MinDistObs}\left(L\right)$  9:if $d_{min}\leq {\mathrm{Dist}}_{safe}$ then return null10:$d_{goal}\leftarrow ∥Q_{new}-Q_{goal}∥$11:$\mathrm{AddNode}(T,Q_{new},\mathrm{parent}=Q_{0},$12:$\mathrm{MinDistObs}=d_{min},\mathrm{DistGoal}=d_{goal}$13:return $Q_{new}$

### 3.5. B-Spline Optimization and Smoothing Technology

To eliminate sharp inflection points and local discontinuous segments in the initial polyline path, this paper performs interpolation densification on the path points under the premise of satisfying collision-free and safety distance constraints and adopts a B-spline curve for fitting to obtain a continuous, smooth and trackable trajectory.

Given the maximum allowable spacing $L_{\mathrm{int},\max}$ between adjacent control points, when the Euclidean distance $\mathrm{Dist}(Q_{i},Q_{i+1})$ between two adjacent points is greater than $L_{\mathrm{int},\max}$, intermediate control points are inserted in the interval $\left[Q_{i},Q_{i+1}\right]$, and the number of inserted points is:

(36) $$n_{\mathrm{int}}=\max\;\left(0,\left\lceil \frac{\mathrm{Dist}(Q_{i},Q_{i+1})}{L_{\mathrm{int},\max}}\right\rceil -1\right)$$

where ⌈·⌉ is the ceiling function. The *k*-th interpolation point is obtained by linear interpolation:

(37) $$Q_{i,k}=Q_{i}+\frac{k}{n_{\mathrm{int}}+1}\;\left(Q_{i+1}-Q_{i}\right),k=1,\ldots ,n_{\mathrm{int}}$$

When $n_{\mathrm{int}}=0$, no intermediate points are inserted, and only the endpoints $Q_{i},Q_{i+1}$ are retained. The set of control points ${\left\{Q_{j}\right\}}_{j=1}^{M}$ is obtained after interpolation, and an *m*-order B-spline is used for geometric smoothing of the path. Let the non-decreasing node vector be $T={\left\{t_{j}\right\}}_{j=1}^{M+m}$, and the first-order basis function is:

(38) $$B_{j,1}\left(\tau \right)=\left\{\begin{array}{c} 1,\tau \in [t_{j},t_{j+1})\\ 0,\;\;\mathrm{else} \end{array}\right.$$

The high-order basis function is derived by the Cox-deBoor recursion:

(39) $$B_{j,m}\left(\tau \right)=\frac{\tau -t_{j}}{t_{j+m-1}-t_{j}}B_{j,m-1}\left(\tau \right)+\frac{t_{j+m}-\tau }{t_{j+m}-t_{j+1}}B_{j+1,m-1}\left(\tau \right)$$

If the denominator is 0, the coefficient of the corresponding term is set to 0. In the parameter interval $\tau \in [t_{m},t_{M+1}]$, the smooth trajectory Γ(τ)=(X(τ),Y(τ)) is:

(40) $$\Gamma \left(\tau \right)=\sum_{j=1}^{M}Q_{j}B_{j,m}\left(\tau \right)$$

where *M* is the number of control points and $Q_{j}$ is the *j*-th control point. Due to the convex hull property of B-spline, the trajectory point corresponding to any τ satisfies:

(41) $$\Gamma \left(\tau \right)\in \mathrm{conv}\left\{Q_{1},Q_{2},\ldots ,Q_{M}\right\}$$

where conv(·) denotes the convex hull of the control point set. To ensure that the smoothed path still satisfies the collision-free and safety distance constraints, collision detection is performed on the smoothed curve; if a collision occurs, iterative processing is conducted by further densifying control points, reducing the order or falling back to the original polyline path until the constraints are satisfied.

Furthermore, to adapt to different path characteristics, the key parameters are set adaptively:

(1) Maximum segment length setting

The maximum segment length is set according to the total path length $S_{\mathrm{total}}$, the segment adjustment coefficient $k_{\mathrm{seg}}$ and the basic segment length upper limit $L_{\mathrm{base}}$:

(42) $$L_{\mathrm{seg},\max}=L_{\mathrm{base}}+\frac{S_{\mathrm{total}}}{k_{\mathrm{seg}}}$$

A longer path can appropriately increase the segment length to reduce the number of segments; a larger $k_{\mathrm{seg}}$ results in a smaller segment length, thus improving the local fitting accuracy and smoothness.

(2) Adaptive selection of B-spline order

The B-spline order is adaptively selected according to the number of control points:

(43) $$m=\left\{\begin{array}{c} \min\left(m_{1},M\right),\;\;\;\;M<M_{th},\\ \min\left(m_{2},M\right),\;\;\;\;M\geq M_{th},\\ \min\left(m_{2},M\right),\;\;\;\;special_{path}, \end{array}\right.$$

where $M_{th}$ is the control point threshold; $m_{1}$ and $m_{2}$ correspond to short and long paths respectively; and $special_{path}$ denotes scenarios such as narrow passages and sharp turns, where the order is reduced to suppress local oscillation.

(3) Adaptive parameter sampling point sequence construction

To improve the discrete resolution in high-curvature regions, an adaptive parameter sampling point sequence is constructed:

(44) $$N_{\mathrm{ref}}=\left[M\left(1+k_{\psi }\kappa_{\max}\right)\right],\tau_{\mathrm{ref}}=\mathrm{linspace}\left(t_{m},t_{M+1},N_{\mathrm{ref}}\right)$$

where $\kappa_{\max}$ is the maximum curvature of the path, $k_{\psi }$ is the curvature sensitivity coefficient, and linspace(·) denotes equidistant sampling in the interval. A larger curvature or a higher $k_{\psi }$ results in denser sampling, thus obtaining a trajectory with smoother dynamic changes in the turning region.

**Figure 6:**
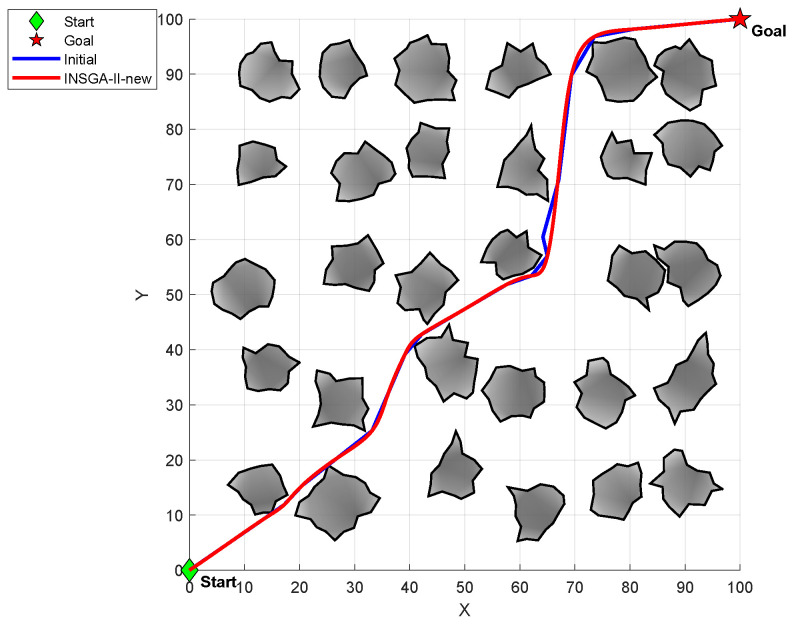
Comparison diagram of paths before and after B-spline optimization.

In summary, this paper improves the continuity and smoothness of the trajectory through interpolation densification, B-spline fitting and adaptive parameter adjustment, and naturally connects with the path generated by the aforementioned expansion strategies, providing a more kinematically constrained trackable trajectory for mobile robots. The comparison of paths before and after B-spline optimization is shown in Figure 6.

### 3.6. Basis for Parameter Setting and Sensitivity Discussion

BGSE-RRT* involves parameters such as thresholds, probabilities, step sizes and weights. To avoid environment-specific “manual parameter tuning”, this paper reuses the same set of parameters and constraints for repeated comparisons in six simulation environments and ROS/real-robot joint validation and explains the parameters in groups according to “sensitivity –meaning –influence direction”. Among them, $\Omega_{s}\left(D\right)$ is the bi-tree cooperative switching probability function (adjusting the rhythm of exploration/convergence), and Ω is the multi-objective evaluation weight (only affecting the sorting of candidate points without changing the collision/safety feasibility check).

(1) Safety and Feasibility Parameters (High Sensitivity, Hard Constraints)

Including $Dist_{safe}$, boundary constraints and collision criteria: The expansion edge is directly rejected if it collides with obstacles or the safety distance is insufficient. An increase in $Dist_{safe}$ improves the safety margin but compresses the feasible space and may increase the planning time; a decrease in $Dist_{safe}$ has the opposite effect but reduces the safety redundancy. Such parameters are determined by the robot size, sensing error and safety boundary and can be transferred across scenarios.

(2) Cooperative Switching and Convergence Parameters (Medium Sensitivity, Stage Control)

The switching probability Ω is given by the piecewise function $\Omega_{s}\left(D\right)$ driven by distance feedback, which is only determined by a small number of scale parameters including $\Omega_{min}$, $\Omega_{max}$ and $D_{th}$: Ω tends to $\Omega_{max}$ as *D* decreases to enhance convergence and takes $\Omega_{min}$ when $D>D_{th}$ to maintain exploration; $t_{iter,min}$ is used to suppress early jitter. The target binding threshold $L_{th}$, adaptive step size and direction normalization further reduce the scale sensitivity.

(3) Multi-objective Weights and Post-processing (Low Sensitivity, Preference Items; Including Safety Distance Rechecking)

Normalized multi-objective evaluation is adopted for multi-sector expansion, and a unified weight vector $\omega ={\left[0.4,0.15,0.3,0.15\right]}^{T}$ is used in this paper; ω is only used to sort the candidate points that pass the collision/safety screening and usually does not change the feasibility. B-spline smoothing is set adaptively, and collision and safety distance rechecking are performed after smoothing. If necessary, densification/reduction in order/fallback are adopted to ensure that the final trajectory is safe and feasible.

In summary, this paper organizes the parameters with a “three-layer desensitization” mechanism: hard constraints ensure feasibility, medium-sensitivity parameters analytically control exploration/convergence, and the remaining parameters are desensitized through normalization/adaptation and safety distance rechecking; combined with the reuse of unified parameters, it can be shown that the performance gain mainly comes from structural and mechanism design rather than manual parameter tuning.

## 4. Experiments and Results

### 4.1. Experimental Setup

To evaluate the planning performance of the proposed BGSE-RRT* in complex static planar environments, we designed five simulation environments and one ROS/real-robot joint validation environment and conducted 200 trials under the same parameter set and constraints, comparing BGSE-RRT* with GB-RRT*, BI-RRT*, BI-APF-RRT*, and BAI-RRT*.

A 100 m × 100 m two-dimensional simulation platform was implemented in MATLAB R2024a, and additional ROS and real-robot experiments were performed. The evaluation metrics included planning time, path length, safety distance, steering-angle RMS, iteration count, and number of nodes. Environments 1–3 were dense small-obstacle environments; Environments 4–5 were narrow-passage environments formed by large obstacles; and Environment 6 was the ROS and real-robot joint verification environment.

**Figure 7:**
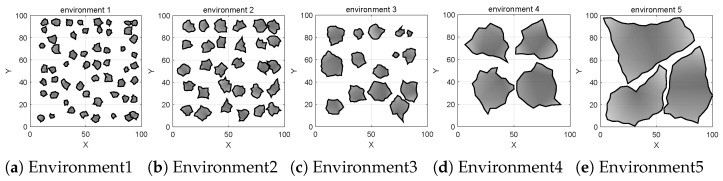
Five simulation environments.

**Figure 8:**
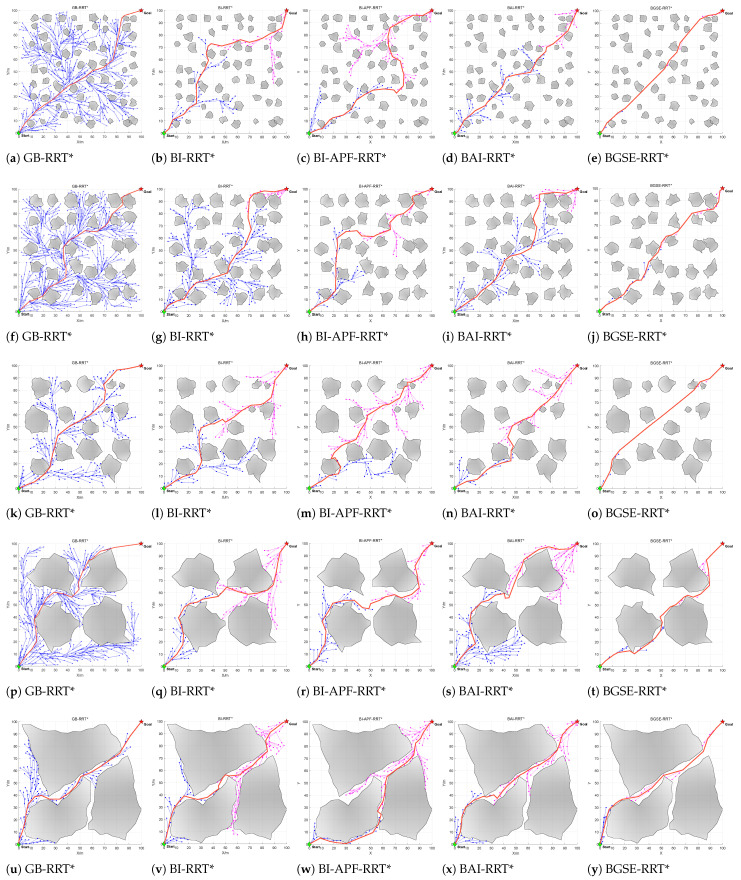
Running results of five algorithms.

**Figure 9:**
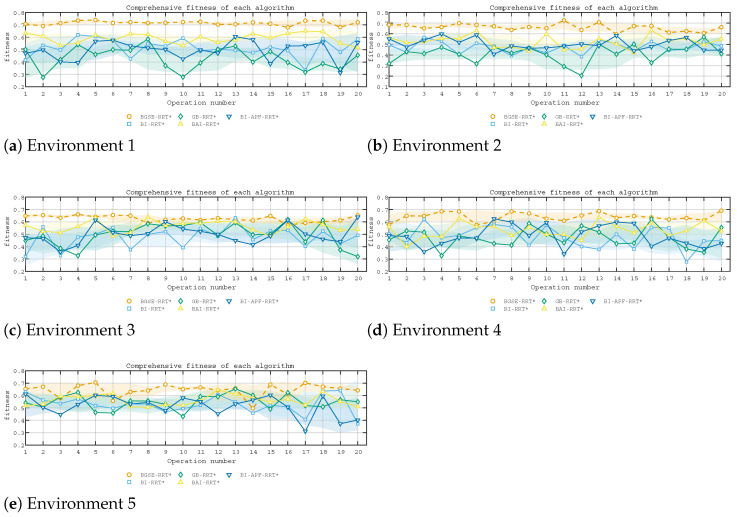
Weighted fitness curves of five algorithms in Environments 1–5.

**Figure 10:**
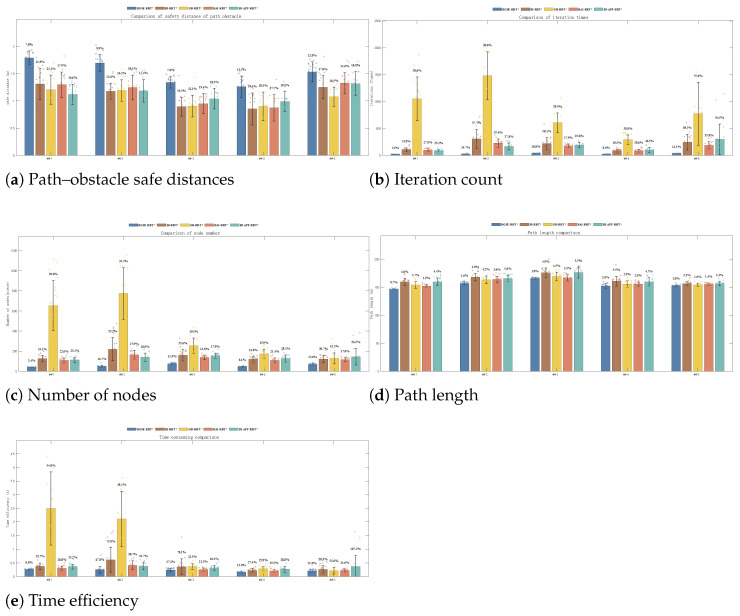
Average values of 200 experiments for five algorithms in Environments 1–5.

Environments 1–5 are shown in Figure 7. The qualitative comparisons in Environments 1–5 are shown in Figure 8, the weighted fitness curves are shown in Figure 9, and the averaged results over 200 trials are summarized in Figure 10 and Table 1.

#### 4.1.1. Dense Small-Obstacle Environments

Planning time: Compared with GB-RRT*, BI-RRT*, BI-APF-RRT*, and BAI-RRT*, BGSE-RRT* reduces planning time by 84.71%, 43.70%, 28.97%, and 22.45%, respectively. This improvement mainly comes from: (i) the bi-tree cooperative adaptive switching, which adjusts the goal bias strength according to the inter-tree distance and the search stage, and (ii) the fact that when goal-guided expansion is blocked, the local multi-sector refined expansion advances via comprehensive candidate evaluation, thereby reducing invalid expansion and strengthening obstacle avoidance.

Path length: Compared with GB-RRT*, BI-RRT*, BI-APF-RRT*, and BAI-RRT*, BGSE-RRT* shortens path length by 3.72%, 6.88%, 6.58%, and 2.94%, respectively. With adaptive switching, global exploration is preserved at long inter-tree distances, while at short distances the expansion target is switched to the nearest connectable node in the opposite tree, guiding both trees to grow along a shorter connection direction. Meanwhile, goal-guided expansion accelerates tree convergence, and the multi-sector mechanism suppresses detour redundancy when blocked.

Safety distance: Compared with GB-RRT*, BI-RRT*, BI-APF-RRT*, and BAI-RRT*, BGSE-RRT* increases safety distance by 48.33%, 44.81%, 46.11%, and 39.43%, respectively. This gain is attributed to: (i) explicit safety constraints and direction correction in the multi-constraint feasibility check during goal-guided expansion; (ii) the safety term in the multi-sector candidate evaluation; and (iii) collision and safety distance rechecking after B-spline smoothing, which maintains the safety margin.

In addition, BGSE-RRT* also reduces iteration count and number of nodes, resulting in a more compact tree and stronger trackability.

#### 4.1.2. Large-Obstacle Occluded Environments

Planning time: Compared with GB-RRT*, BI-RRT*, BI-APF-RRT*, and BAI-RRT*, BGSE-RRT* reduces planning time by 30.36%, 31.53%, 7.35%, and 33.94%, respectively, consistent with the advantages discussed above.

Path length: Compared with GB-RRT*, BI-RRT*, BI-APF-RRT*, and BAI-RRT*, BGSE-RRT* reduces path length by 1.52%, 3.85%, 3.54%, and 1.95%, respectively. Dynamic target binding guides the two trees to advance along a shorter corridor connection direction and the selection mechanism stabilizes feasible detours when blocked, yielding paths closer to passable regions.

Safety distance: Compared with GB-RRT*, BI-RRT*, BI-APF-RRT*, and BAI-RRT*, BGSE-RRT* increases safety distance by 20.68%, 15.32%, 9.58%, and 10.42%, respectively. When blocked, the multi-sector mechanism prioritizes safer directions with a feasibility check, and together with multi-constraint collision checks and the B-spline convex hull property, the trajectory shape remains safe.

BGSE-RRT* also reduces iteration count and number of nodes, achieving a better balance between exploration efficiency and path quality in narrow-passage environments.

#### 4.1.3. ROS and Real-Robot Joint Verification

The experiments are conducted in a 20 m × 40 m ROS simulation environment and a 10 m × 20 m real field site. The real-robot platform is a LanderPi Mecanum mobile robot. Based on ROS, chassis control is realized by sending linear velocity *v* and angular velocity ω via topic $/cmd_{vel}$. To match the two-dimensional planar kinematic model in this paper, only the (v,ω) channels are used with lateral velocity fixed to $v_{y}=0$, and the bounds |v|≤0.35 m/s and |ω|≤1.80 rad/s are applied for feasibility check and time calibration. Key robot parameters are: wheel diameter 0.060 m, wheelbase 0.150 m, track width 0.160 m, body size 0.212 × 0.174 × 0.441 m, and mass 1.79 kg. Sensors include a 2D LiDAR (MS200, 360°, angular resolution 0.8°, 7–15 Hz, range 0.1–13.4 m), an IMU (QMI8658), and an AB-phase Hall encoder (13 poles, 1:20). Computation and communication use a Raspberry Pi 5 (Raspberry Pi OS, ROS1 Melodic deployed in Docker). The chassis controller is an STM32 F407VET6 communicating with ROS via serial/USB-serial. The ROS simulation uses the same planar model and the same velocity/angular-velocity constraints as the real robot to ensure the consistency and reproducibility of joint verification.

**Figure 11:**
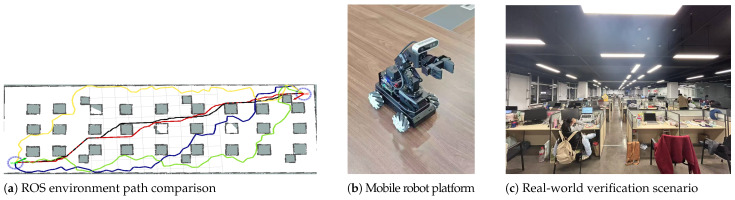
ROS and real-robot joint verification results.

The ROS path comparison shows that BGSE-RRT* (black) achieves the best overall path quality; GB-RRT* (yellow) produces denser corners and higher redundancy, while BGSE-RRT* outperforms BI-RRT* (green), BI-APF-RRT* (red), and BAI-RRT* (blue). Compared with these methods, BGSE-RRT* reduces the average planning time by 30.68%, shortens the average path length by 4.15%, increases the average safety distance by 32.20%, reduces the average steering-angle RMS by 18.64%, reduces the average iteration count by 90.46%, and reduces the average number of nodes by 67.21%. In real-robot navigation, a single run takes about 26 s with a 100% trajectory-tracking success rate, indicating that the proposed method can generate shorter and safer navigation paths. The joint verification results are shown in Figure 11.

## 5. Conclusions

BGSE-RRT* establishes a bi-tree cooperative adaptive switching mechanism to achieve a dynamic balance between global exploration and local convergence during searching. Under a multi-constraint feasibility check, it adopts goal-guided expansion to accelerate bi-tree convergence and improve connection efficiency. When goal-guided expansion is blocked, BGSE-RRT* switches to local multi-sector refined expansion, generating candidate expansion points and advancing through selection to enhance obstacle avoidance. If the multi-sector mechanism still fails, sampling-point-guided expansion is activated to suppress invalid exploration and maintain forward progress until a feasible path is found. Finally, B-spline smoothing is applied to improve trajectory continuity.

With these complementary mechanisms, BGSE-RRT* achieves up to an 84.71% reduction in planning time in dense small-obstacle environments, with shorter paths and 39–48% increases in safety distance. In narrow-passage environments, planning time is reduced by 7–34% and safety distance is increased by 10–21%. In ROS and real-robot joint verification, the average planning time is reduced by 30.68%, the path is safer and easier to track, and both iteration count and number of nodes are reduced; a real-robot run takes about 26 s with a 100% trajectory-tracking success rate.

This work primarily targets two-dimensional static planar environments, and systematic extensions to dynamic obstacles and multi-robot cooperative planning remain to be developed. Although the algorithm contains multiple threshold and probability parameters, the risk of manual tuning has been mitigated by using a unified parameter set across all environments and by providing a hierarchical sensitivity discussion; nevertheless, stronger adaptivity and broader verification are still needed under more complex tasks and platform constraints.

Future work will introduce motion prediction and online replanning with explicit evaluation of real-time performance and safety. We will also extend the framework to multi-robot cooperative planning by integrating task allocation and collision-avoidance constraints and will further generalize the goal-guided and multi-sector selection mechanisms to higher-dimensional problems by combining them with spatiotemporal trajectory optimization.

## Figures and Tables

**Table 1:** Averages of five algorithm results over 200 experiments in Environments 1–5.

Environment	Algorithm	Path Length /m	Iteration Count/Piece	Number of Nodes /Piece	Safe Distance /m	Planning Time /s	Coefficient of Variation/%
Environment 1	GB-RRT*	154.16	1052.00	653.25	1.20	2.49	31.28
	BI-RRT*	159.57	107.95	125.90	1.31	0.38	22.90
	BI-APF-RRT*	159.58	87.15	113.50	1.12	0.37	18.90
	BAI-RRT*	152.09	96.05	107.85	1.30	0.30	19.24
	BGSE-RRT*	**146.13**	**19.75**	**46.00**	**1.85**	**0.26**	**4.52**
Environment 2	GB-RRT*	163.26	1482.50	773.60	1.19	2.11	26.42
	BI-RRT*	167.82	307.65	220.05	1.17	0.61	40.06
	BI-APF-RRT*	165.67	167.20	139.45	1.18	0.38	24.28
	BAI-RRT*	163.92	227.40	163.15	1.25	0.42	24.74
	BGSE-RRT*	**156.80**	**24.10**	**49.55**	**1.69**	**0.26**	**18.94**
Environment 3	GB-RRT*	169.12	607.55	254.95	0.90	0.37	21.46
	BI-RRT*	175.70	220.25	159.45	0.89	0.36	37.60
	BI-APF-RRT*	176.19	193.85	154.95	1.04	0.32	19.58
	BAI-RRT*	166.62	179.10	136.70	0.95	0.26	16.34
	BGSE-RRT*	**165.53**	**38.9**	**74.7**	**1.34**	**0.24**	**11.74**
Environment 4	GB-RRT*	155.44	289.80	173.70	1.30	0.90	23.16
	BI-RRT*	160.56	88.20	118.80	1.23	0.85	24.20
	BI-APF-RRT*	159.44	100.80	126.25	1.30	0.99	29.16
	BAI-RRT*	155.45	89.65	109.10	1.27	0.87	19.72
	BGSE-RRT*	**152.10**	**22.10**	**45.85**	**1.33**	**1.26**	**9.96**
Environment 5	GB-RRT*	154.03	773.35	128.80	1.07	0.22	38.18
	BI-RRT*	156.41	247.60	121.70	1.25	0.26	31.90
	BI-APF-RRT*	156.49	299.65	144.70	1.31	0.37	55.46
	BAI-RRT*	155.36	187.45	115.25	1.32	0.22	18.80
	BGSE-RRT*	**152.66**	**33.45**	**67.90**	**1.53**	**0.20**	**12.38**

## Data Availability

The data presented in this study are available on request from the corresponding author.
